# Additional Use of Prostacyclin Analogs in Patients With Pulmonary Arterial Hypertension: A Meta-Analysis

**DOI:** 10.3389/fphar.2022.817119

**Published:** 2022-02-09

**Authors:** Pengwei Wang, Jiaxin Deng, Quanying Zhang, Hongyan Feng, Yongheng Zhang, Yizhong Lu, Lizhu Han, Pengfei Yang, Zhijian Deng

**Affiliations:** ^1^ Department of Pharmacy, The First Affiliated Hospital of Xinxiang Medical University, Weihui, China; ^2^ Department of Endoscopic Surgery, The Sixth Affiliated Hospital of Sun Yat-sen University, Guangzhou, China; ^3^ Nursing Department, The First Affiliated Hospital of Xinxiang Medical University, Weihui, China; ^4^ Outpatient Department, The First Affiliated Hospital of Xinxiang Medical University, Weihui, China; ^5^ Department of Pharmacy, Sichuan Academy of Medical Sciences and Sichuan Provincial People’s Hospital, School of Medicine, University of Electronic Science and Technology of China, Chengdu, China; ^6^ Henan International Joint Laboratory of Cardiovascular Remodeling and Drug Intervention, Xinxiang Key Laboratory of Vascular Remodeling Intervention and Molecular Targeted Therapy Drug Development, College of Pharmacy, Xinxiang Medical University, Xinxiang, China

**Keywords:** pulmonary arterial hypertension, prostacyclin analogs, combination therapy, meta-analysis, clinical worsening, adverse events

## Abstract

**Background:** Combination therapy has become an attractive option in pulmonary arterial hypertension (PAH) treatment. The aim of this study was to investigate whether additional use of prostacyclin analogs could exert any additional benefits over background targeted therapies in PAH patients.

**Methods:** Searches were performed on PubMed, Embase, and ClinicalTrials.gov from inception to 1 October 2021. Randomized controlled trials were included if patients had been treated with prostacyclin analog-containing combination therapy and compared with the use of other PAH-specific background therapies. The bias risk and statistical analysis of the enrolled studies were performed with RevMan 5.1. Sensitivity analysis and funnel plot were used to evaluate the stability and publication bias, respectively. PROSPERO registered number CRD42021284196.

**Results:** Ten trials involving 1828 patients were included. Prostacyclin analog treatment was associated with greater improvement in clinical worsening (risk ratio [RR], 0.70; 95% confidence interval [CI], 0.57–0.86), 6-min walk distance (mean difference [MD], 37.17 m; 95% CI, 3.01–71.33 m), NYHA/WHO functional class (RR, 1.58; 95% CI, 1.21–2.05), mean pulmonary artery pressure (MD, −9.23 mmHg; 95% CI, −17.44 to −1.03 mmHg), and cardiac index (MD, 0.41 L/min/m^2^; 95% CI, 0.26–0.55 L/min/m^2^) than the control group. No significant differences in pulmonary vascular resistance (MD, −137.22 dyn·s/cm^5^; 95% CI, −272.61 to −1.84 dyn·s/cm^5^) and all-cause mortality (RR, 0.96; 95% CI, 0.57–1.61) were found between the prostacyclin analog group and control group. Of note, more adverse events (RR, 1.07; 95% CI, 1.02–1.13) occurred in the prostacyclin analog group but no significant increase in serious adverse events (RR, 1.25; 95% CI, 0.75–2.11).

**Conclusion:** Additional prostacyclin analog treatment exerted benefits on clinical worsening, exercise capacity, functional class, mean pulmonary artery pressure, and cardiac index in PAH patients, but it was associated with overall risk of adverse events.

**Clinical Trial Registration:** https://www.crd.york.ac.uk/prospero/display_record.php?ID=CRD42021284196, identifier CRD42021284196.

## Introduction

Pulmonary arterial hypertension (PAH) is a heterogeneous disorder characterized by progressive remodeling of the pulmonary vasculature and the increase in pulmonary arterial pressure (PAP) and pulmonary vascular resistance (PVR), which is also a devastating and rare disease with an approximated incidence of up to 7.6 cases per million (Peacock et al., 2007; Ali et al., 2021). Without appropriate care, it may carry a poor prognosis.

Based on the current understanding of the pathogenesis of PAH, approved PAH-targeted medications mainly target nitric oxide, endothelin, and prostacyclin pathways (Shivakumar et al., 2020). Theoretically, combining two or more drugs acting on different pathways may have a better therapeutic effect. Prostacyclin, a powerful vasodilator, is synthesized mainly in the vascular endothelium and also possesses antiproliferative and antiplatelet properties (Galiè et al., 2003). Moreover, in PAH, the level of prostacyclin and prostaglandin I_2_ synthase was decreased (Christman et al., 1992; Tuder et al., 1999). Therefore, prostacyclin analogs, such as beraprost, treprostinil, and iloprost, target the prostacyclin pathway, are efficacious for PAH patients, and have been advocated for treating PAH in clinical practice (Thenappan et al., 2018). In addition, the endothelin pathway and nitric oxide pathway are also involved in the pathogenesis of PAH, and drugs targeting these two pathways have been proven to ameliorate exercise capacity, functional class, and clinical worsening (Rubin et al., 2002; Galiè et al., 2005; Ghofrani et al., 2013).

Despite previous meta-analyses supporting the superior effects of PAH-specific combination therapy over monotherapy (Fox et al., 2016; Lajoie et al., 2016), whether prostacyclin analog-containing combination therapy could further exert any additional benefits over background targeted therapies is still unclear. Moreover, some new studies related to our research have been published (Han et al., 2017; Zamanian, 2017; Galiè, 2019; White et al., 2020). Therefore, we performed this meta-analysis to evaluate the efficacy of additional use of prostacyclin analogs in patients with PAH.

## Methods

### Search Strategy

The research adhered to the Preferred Reporting Items for Systematic Reviews and Meta-Analyses (PRISMA) statement (Page et al., 2021). Moreover, it has been registered at PROSPERO (CRD42021284196). We searched PubMed, Embase, and ClinicalTrials.gov for studies referring to prostacyclin analog-containing combination therapy up to 1 October 2021, using the keywords: “pulmonary arterial hypertension” AND (“prostacyclin” OR “treprostinil” OR “iloprost” OR “beraprost” OR “epoprostenol”), not limited by language (Supplementary Table S1).

### Study Selection and Inclusion Criteria

Two reviewers (PW and JD) independently selected eligible studies and dealt with discrepancies by referring with another reviewer (ZD). The inclusion criteria were 1) patients diagnosed with PAH; 2) randomized controlled trials (RCTs) have a minimum follow-up of 12 weeks and reported at least one of the following endpoints: clinical worsening, 6-min walk distance (6 MWD), functional class, mean pulmonary artery pressure (mPAP), cardiac index, PVR, all-cause mortality, and adverse events; and 3) RCTs (whether published or unpublished) assessing the effectiveness of prostacyclin analog-containing combination therapy compared to other PAH-specific background therapies. Conference abstracts can also be included if they reported the relevant outcome. Duplicated publications or self-control studies were excluded.

### Data Extraction

Two reviewers (PW and QZ) independently extracted related data using an extraction form and consulted another author (PY) to resolve discrepancies. Extracted data included first author, publication year, demographics, study design, interventions, outcome measures, and adverse events. When standard deviation was not directly available in the study, it was calculated from standard error, confidence interval (CI), or *p* value.

### Quality Assessment

The quality of each study was assessed using Cochrane-recommended tools including the domains of selection bias, performance bias, detection bias, attrition bias, reporting bias, and other bias (Higgins et al., 2011).

### Statistical Analysis

Categorical outcomes (clinical worsening, all-cause mortality, NYHA/WHO functional class, adverse events, etc.) and continuous outcomes (6 MWD, mPAP, cardiac index, and PVR) were presented as risk ratio (RR) with 95% confidence interval (CI) and mean difference (MD) with 95% CI, respectively. Heterogeneity was measured using *I* ^*2*^ [*I* ^*2*^ > 50% indicating significant heterogeneity (Higgins and Thompson, 2002)]. If *I* ^*2*^ > 50%, the random-effects model was applied. Otherwise, the fixed-effects model was used. When possible, subgroup analysis based on NYHA/WHO functional class, PAH etiology, and background targeted therapies was performed. Sensitivity analyses were carried out to study the robustness of our results to two assumptions that only included the double-blind studies including the studies with a sample size more than 30 or excluding the unpublished studies. Publication bias was evaluated by funnel plot. All analyses were performed with RevMan (version 5.1.4).

## Results

### Study Selection and Characteristics

**FIGURE 1:**
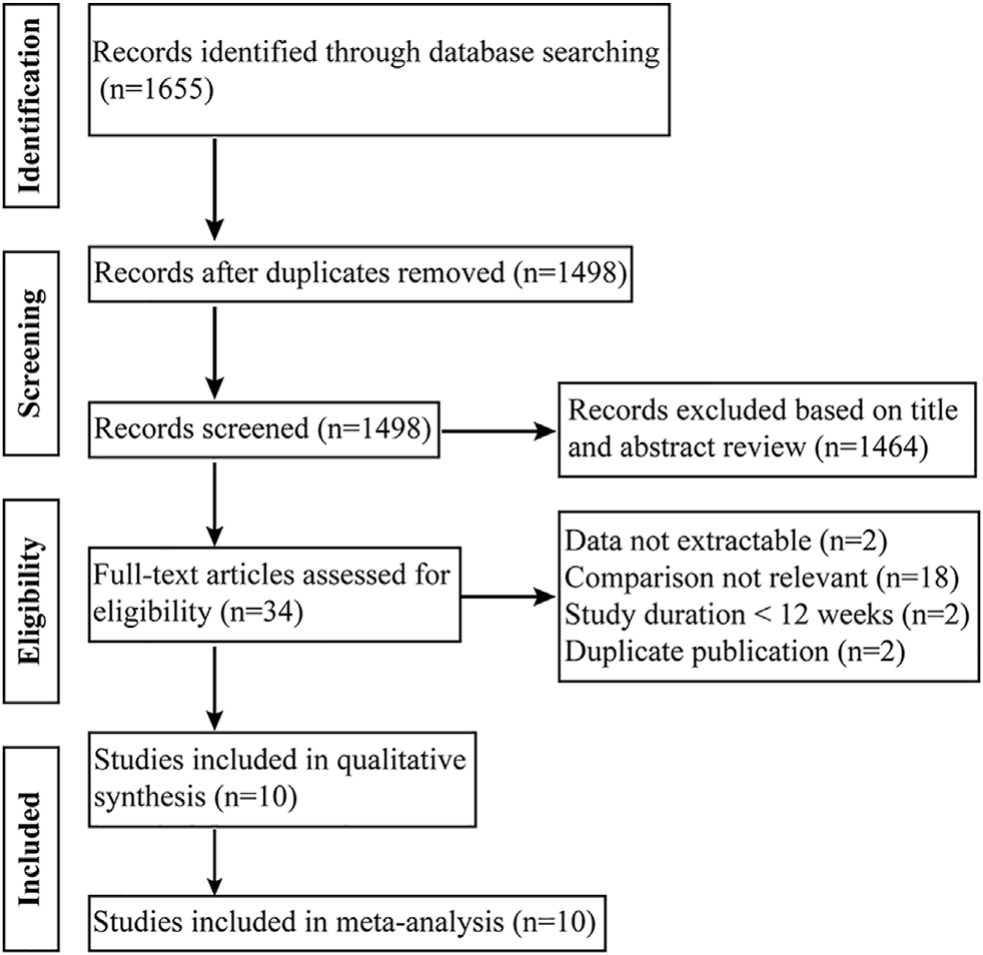
Flowchart of study identification, inclusion, and exclusion.

A total of 1,655 articles were identified by searching three databases, and 157 duplicates were removed, leaving 1,498 studies for screening (Figure 1). Ultimately, 10 RCTs including 1828 patients met the inclusion criteria (Hoeper et al., 2006; Mclaughlin et al., 2006; Mclaughlin et al., 2010; Tapson et al., 2012; Jing et al., 2013; Tapson et al., 2013; Han et al., 2017; Zamanian, 2017; Galiè, 2019; White et al., 2020), of which 920 received prostacyclin analog-containing combination therapy and 908 received other PAH-specific background therapies. In 10 studies, all patients were receiving PAH-specific treatment. The majority of the patients enrolled were female (79%) and were in NYHA/WHO functional class II or III. The characteristics and bias risk analysis of the enrolled studies are presented in Table 1 and Supplementary Figure S1, respectively. Among the 10 included studies, 7 were double-blind, 2 were open-label, and the blinding method was unclear for 1 study. The main high risks of bias were performance bias and detection bias.

**TABLE 1:** Basic characteristics of included studies.

Author (year)	Major participants	N	F (%)	Follow-up	Etiology (%)	NYHA/WHO functional class (%)	Baseline therapy	Therapeutic arm	Study design
Galiè (2019)	North American	40	82.5	16 weeks	IPAH/FPAH (100%)	I (NA), II (NA), III (NA), IV (NA)	Sildenafil or sildenafil + bosentan	Inhaled iloprost 5 μg 6/day	R, DB, MC, PC
Han et al. (2017)	Asian	15	66.7	3 months	IPAH (80%), CTEPH (20%)	III (66.7%), IV (33.3%)	Bosentan	Inhaled iloprost 10 μg 4–6/day	R, OL
Hoeper et al. (2006)	European	40	77.5	12 weeks	IPAH (100%)	III (100%)	Bosentan	Inhaled iloprost 5 μg 6/day	R, OL, MC
Jing et al. (2013)	Asian	60	76.7	24 weeks	IPAH/FPAH (55%), APAH (45%)	II (45%), III (53.3%), IV (1.7%)	Sildenafil	Oral beraprost	R
Mclaughlin et al. (2006)	North American	67	79	12 weeks	IPAH (55%), APAH (45%)	II (1.5%), III (94%), IV (4.5%)	Bosentan	Inhaled iloprost 5 μg 6–9/day	R, DB, MC, PC
Mclaughlin et al. (2010)	North American	235	81.3	12 weeks	IPAH/FPAH (56%), APAH (33%), others (11%)	III (98%), IV (2%)	Bosentan or sildenafil	Inhaled treprostinil 18–54 μg 6/day	R, DB, MC, PC
Tapson et al. (2012)	North American	350	82.3	16 weeks	IPAH/FPAH (66%), APAH (34%)	I (0.9%), II (20.6%), III (76%), IV (2.6%)	ERA, PDE5i, or both	Oral treprostinil 0.5–16 mg bid	R, DB, MC, PC
Tapson et al. (2013)	North American	310	77.7	16 weeks	IPAH/FPAH (65%), APAH (35%)	II (25.8%), III (72.6%), IV (1%)	ERA, PDE5i, or both	Oral treprostinil 0.25–10.5 mg bid	R, DB, MC, PC
White et al. (2020)	North American	690	78.8	NA	IPAH/HPAH (63%), APAH (34%), others (3%)	I (3.2%), II (62.8%), III (33.9%), IV (0.1%)	ERA, PDE5i, or sGCS	Oral treprostinil	R, DB, MC, PC
								0.125–12 mg tid	
Zamanian (2017)	North American	21	76.2	48 weeks	IPAH/HPAH (NA), APAH (NA)	II (NA), III (NA)	Tadalafil	Inhaled treprostinil 18–54 μg qid	R, DB, MC

N, number of patients; F, female; IPAH, idiopathic pulmonary arterial hypertension; CTEPH, chronic thromboembolic pulmonary hypertension; FPAH, familial pulmonary arterial hypertension; APAH, associated pulmonary arterial hypertension; HPAH, heritable pulmonary arterial hypertension; ERA, endothelin receptor antagonist; PDE5i, phosphodiesterase type 5 inhibitor; sGCS, soluble guanylate cyclase stimulator; NA, not available; R, randomized; OL, open-label; DB, double-blind; MC, multicenter; PC, placebo-controlled.

### Efficacy Endpoints

**FIGURE 2:**
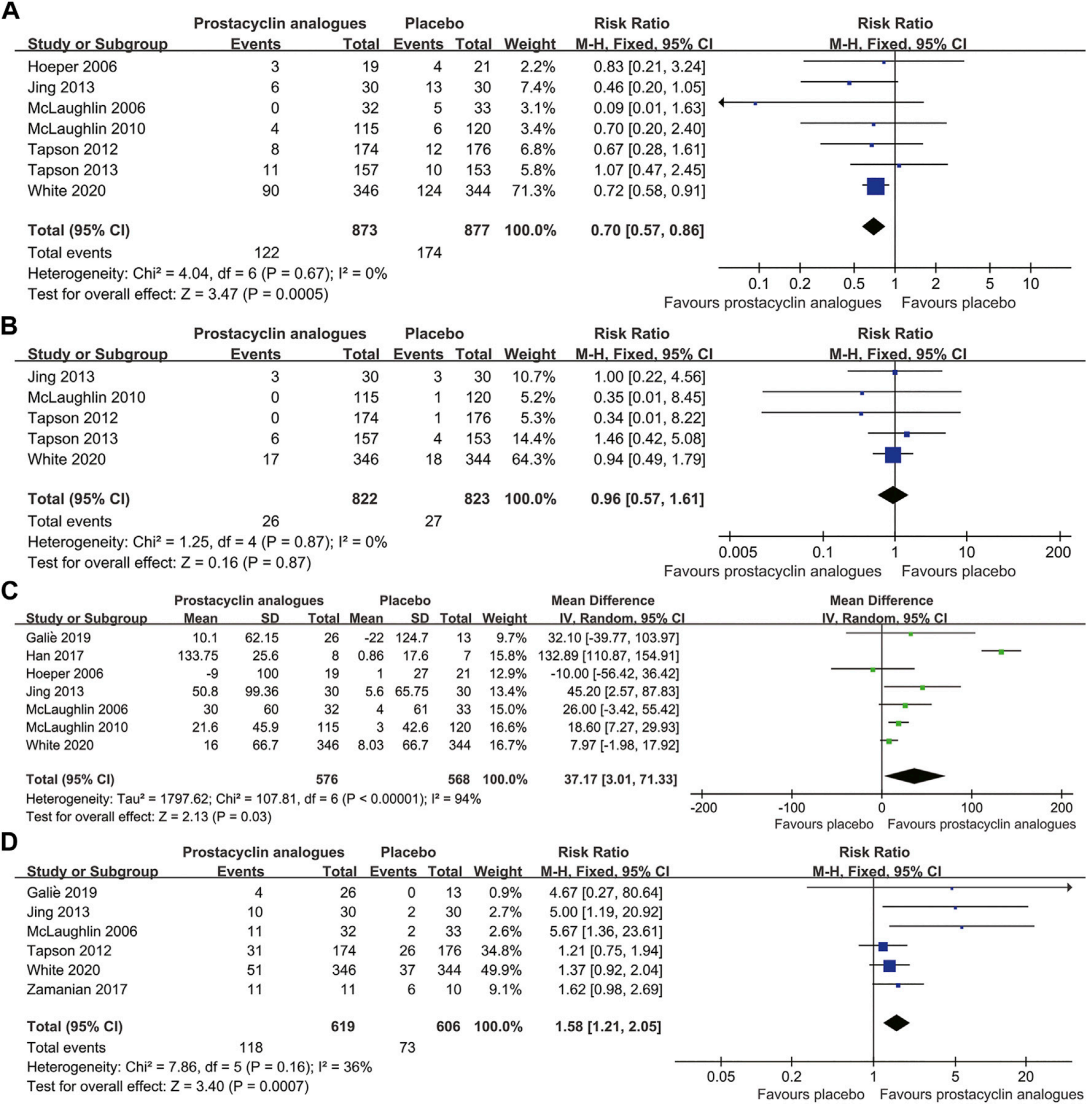
Forest plot comparing prostacyclin analog group with control group for clinical worsening **(A)**, all-cause mortality **(B)**, 6-min walk distance **(C)**, and NYHA/WHO functional class **(D)**.

Among the 1750 subjects in 7 studies, 296 (16.9%) showed clinical worsening, including 174 (19.8%) in the control group and 122 (14.0%) in the prostacyclin analog group. Clinical worsening incidence was significantly lower in the prostacyclin analog group than in the control group (RR, 0.70; 95% CI, 0.57–0.86; *p* < .001), without significant heterogeneity (*I* ^*2*^ = 0%) (Figure 2A).

All-cause mortality in the five trials was 3.2% (53/1,645 patients). Mortality in the prostacyclin analog group and control group was 3.2% (26/822 patients) and 3.3% (27/823 patients), respectively. We found no difference in all-cause mortality between the prostacyclin analog and control groups (RR, 0.96; 95% CI, 0.57–1.61; *p* = .87), with no heterogeneity between studies (*I* ^*2*^ = 0%) (Figure 2B).

Seven RCTs, involving 1,144 patients, compared the changes of 6 MWD in the prostacyclin analogs and control groups. Compared to the control group, the prostacyclin analog group significantly improved 6 MWD by 37.17 m (95% CI, 3.01–71.33 m; *p* = .03), with extreme heterogeneity (*I* ^*2*^ = 94%) (Figure 2C).

Six studies reported 191 (15.6%) patients ameliorated NYHA/WHO functional class at least 1 grade, consisting of 118 (19.1%) patients in the prostacyclin analog group and 73 (12.0%) patients in the control group. Functional class amelioration was superior in the prostacyclin analog group than in the control group (RR, 1.58; 95% CI, 1.21–2.05; *p* < .001), with mild heterogeneity (*I* ^*2*^ = 36%) (Figure 2D).

**FIGURE 3:**
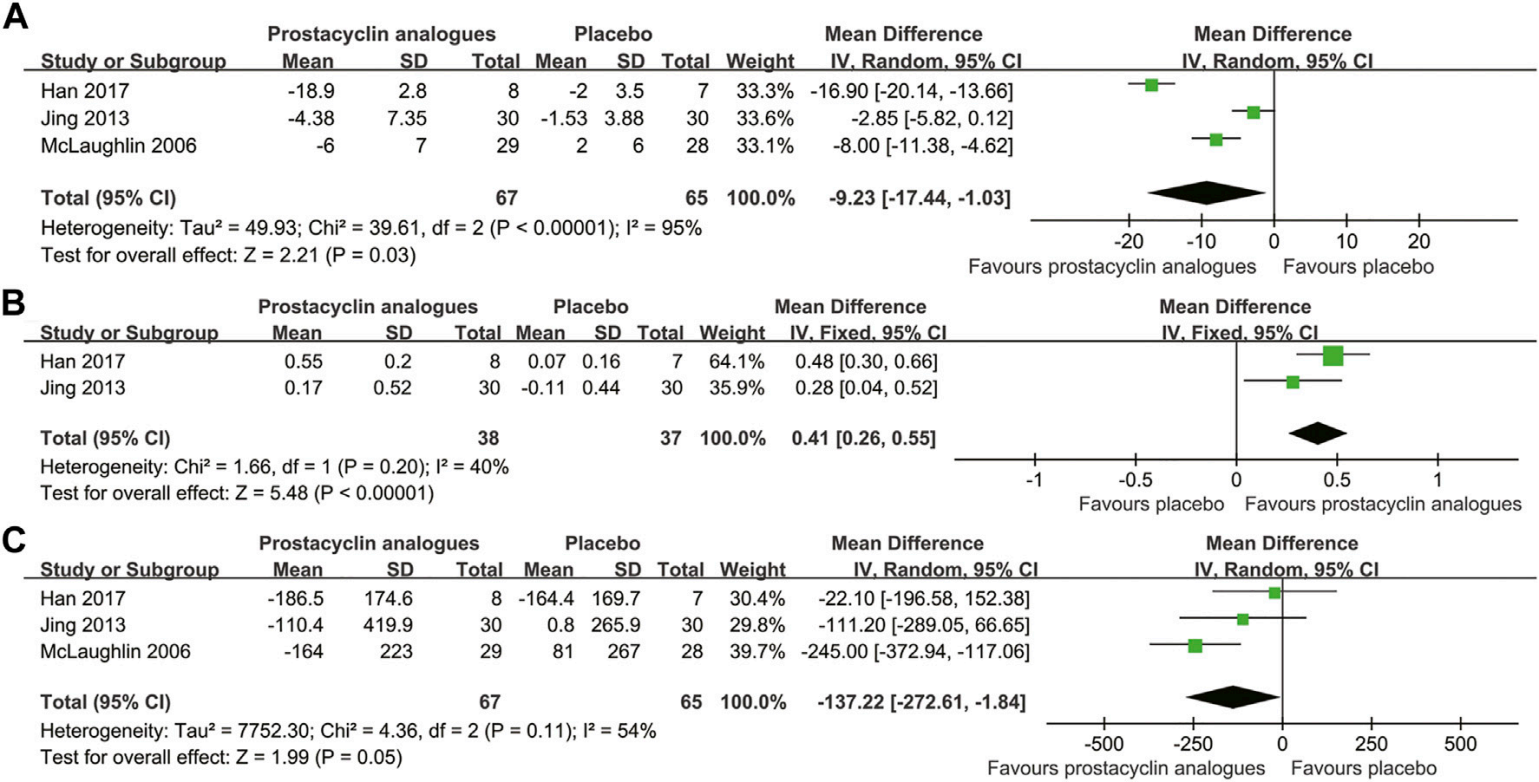
Forest plot comparing prostacyclin analog group with control group for mean pulmonary artery pressure **(A)**, cardiac index **(B)**, and pulmonary vascular resistance **(C)**.

Three studies, involving 132 subjects, compared the changes of mPAP and PVR in the prostacyclin analogs and control groups. Two studies, involving 75 subjects, compared the changes in the cardiac index in the prostacyclin analogs and control groups. Pooled analysis revealed that prostacyclin analogs led to statistically significant reduction in mPAP (MD, −9.23 mmHg; 95% CI, −17.44 to −1.03 mmHg; *p* = .03) (Figure 3A) and obvious increase in the cardiac index (MD, 0.41 L/min/m^2^; 95% CI, 0.26–0.55 L/min/m^2^; *p* < .001) (Figure 3B). However, PVR was improved numerically in the prostacyclin analog group without achieving significance (MD, −137.22 dyn·s/cm^5^; 95% CI, −272.61 to −1.84 dyn·s/cm^5^; *p* = .05) (Figure 3C). There was significant heterogeneity related to the mPAP and PVR measurements among studies (mPAP, *I* ^*2*^ = 95%; PVR, *I* ^*2*^ = 54%). Mild heterogeneity was observed in the cardiac index among studies (*I* ^*2*^ = 40%).

### Safety Endpoints

The analysis indicated that the additional use of prostacyclin analogs significantly increased the overall risk of adverse events (RR, 1.07; 95% CI, 1.02–1.13; *p* = .01). Headache (66.4%), diarrhea (50.6%), and nausea (39.8%) were the three most common adverse events, with risk ratios of 2.16 (95% CI, 1.94–2.40; *p* < .001), 2.63 (95% CI, 2.27–3.05; *p* < .001), and 2.02 (95% CI, 1.73–2.34; *p* < .001), respectively. In addition, flushing, pain in the jaw, vomiting, and pain in extremities were the more obvious increase, with risk ratios of 4.33 (95% CI, 3.46–5.43; *p* < .001), 4.24 (95% CI, 3.12–5.76; *p* < .001), 3.65 (95% CI, 2.12–6.28; *p* < .001), and 2.70 (95% CI, 1.85–3.94; *p* < .001), respectively. Of note, the additional use of prostacyclin analogs did not significantly increase the incidence of serious adverse events (RR, 1.25; 95% CI, 0.75–2.11; *p* = .39). Overall, prostacyclin analog-related adverse drug responses were relatively mild and tolerable. Patients using prostacyclin analogs should pay attention to monitoring adverse reactions.

### Sensitivity Analyses and Publication Bias

Sensitivity analysis was first performed by specifically including double-blind studies, or studies with a sample size more than 30, and almost the same outcomes were seen, except the result of PVR. Second, restricting our analysis to published studies did not alter the pooled results (Supplementary Table S2). A funnel plot showed no obvious asymmetry, indicating minimal publication bias for clinical worsening (Supplementary Figure S2). Moreover, PRISMA 2020 Checklist was used to improve the reporting quality of this meta-analysis (Supplementary Table S3).

## Discussion

This meta-analysis demonstrated the benefits of prostacyclin analog-containing combination treatment in PAH patients. Based on 1,828 patients in 10 studies, we found that additional use of prostacyclin analogs significantly reduce clinical worsening incidence, improve exercise capacity, and ameliorate NYHA/WHO functional class and hemodynamic parameters (mPAP and cardiac index). However, reduction of all-cause mortality and PVR was not detected in this study. Moreover, the prostacyclin analog group was associated with more adverse events than the control group, with no obvious increase in serious adverse events.

Clinical worsening is a composite endpoint, which was used to assess the clinical status and disease progression in PAH patients (Ghio et al., 2021). Additional administration of prostacyclin analogs resulted in alleviated clinical worsening in PAH patients with background targeted therapies. However, 14% of subjects in the prostacyclin analog group still deteriorated into clinical worsening. Moreover, all-cause mortality reduction was not observed in our study, indicating that additional use of prostacyclin analogs could delay disease progression but may not ameliorate the prognosis.

In 7 studies, 6 MWD was used to evaluate the therapeutic efficacy of prostacyclin analogs on exercise capacity in our study (Hoeper et al., 2006; Mclaughlin et al., 2006; Mclaughlin et al., 2010; Jing et al., 2013; Han et al., 2017; Galiè, 2019; White et al., 2020). We found that the MD of the 6 MWD in the prostacyclin analog group was 37.17 m compared with the control group, indicating that additional use of prostacyclin analogs exerted benefits in improving exercise capacity and ameliorating the quality of life of PAH patients. Notably, extreme heterogeneity was detected in 6 MWD among studies. When only four double-blind studies were included, the heterogeneity changed from extreme (*I* ^*2*^ = 94%) to none (*I* ^*2*^ = 0%), and the MD of the 6 MWD in the prostacyclin analog group was 13.60 m compared with the control group.

With regard to functional capacity, we found that prostacyclin analog treatment significantly improved functional capacity as measured by NYHA/WHO functional class. Previous studies had indicated that survival was correlated with NYHA functional class after chronic epoprostenol therapy (NYHA class III/IV versus I/II) (Mclaughlin et al., 2002; Sitbon et al., 2002). In our study, the amelioration of one functional class was seen more often in subjects allocated to prostacyclin analog-containing combination therapy, which provided further evidence and confidence for the clinical efficacy of additional use of prostacyclin analogs in PAH, even if without the reduction of all-cause mortality.

Three meta-analyses had demonstrated the efficacy of prostacyclin in PAH patients (Li et al., 2013; Zheng et al., 2014; Barnes et al., 2019). Of note, their results showed that prostacyclins were efficient in reducing mortality and PVR, which were not consistent with our study. The negative mortality and PVR outcomes in our study may be attributed to the following two reasons: First, the route of drug administration was oral or inhaled in our study. However, the route of drug administration was oral, inhaled, intravenous, or subcutaneous in those three previous meta-analyses (Li et al., 2013; Zheng et al., 2014; Barnes et al., 2019). It is worth noting that the survival benefit was largely due to the subgroup of intravenous preparations in two previous meta-analyses (Zheng et al., 2014; Barnes et al., 2019), while the survival benefit in another meta-analysis (Li et al., 2013) was only due to one intravenous study (Barst et al., 1996). Second, the existence of background targeted therapies already exerted benefits in PAH patients. Therefore, it left no scope for further amelioration on these outcomes. Previous studies did not differentiate between prostacyclin analog monotherapy and prostacyclin analog–containing combination therapy (Li et al., 2013; Zheng et al., 2014; Barnes et al., 2019), while our study focused only on the benefits of additional use of prostacyclin analogs in patients with PAH. Among 4 new studies (Han et al., 2017; Zamanian, 2017; Galiè, 2019; White et al., 2020) added in our study, 2 studies (Han et al., 2017; White et al., 2020) reported the all-cause mortality and the changes of PVR in the prostacyclin analog and control groups. It was found that additional prostacyclin analog treatment did not exert additional benefits on all-cause mortality and PVR in PAH patients, which may be attributed to the previous two reasons.

Our meta-analysis has several limitations. First, most of the participants were in NYHA/WHO class II/III and had a short treatment duration. It might be difficult to detect whether the additional use of prostacyclin analogs could ameliorate all-cause mortality. Second, the studies included in our meta-analysis were heterogeneous in terms of NYHA/WHO functional class, PAH etiology, and background targeted therapies. It is a pity that subgroup analysis based on these aspects was not performed due to limited data. Third, previous studies indicated that patients with intravenous prostacyclins showed improved survival and hemodynamic parameters (Barnes et al., 2019). However, patients included in our study only received oral or inhaled prostacyclin analogs. It was still unknown whether the additional use of intravenous prostacyclin analogs or subcutaneous prostacyclin analogs could exert additional benefits in PAH patients and alter the direction or magnitude of the results in our meta-analysis. Fourth, among the 10 included studies, there were a limited number of clinical studies that have evaluated endpoints such as mPAP (*n* = 3), cardiac index (*n* = 2), PVR (*n* = 3), and serious adverse events (*n* = 3). Therefore, cautious interpretations are needed when considering the effects of additional use of prostacyclin analogs on these endpoints.

Considering the limitations mentioned previously, future RCTs should be designed to assess the long-term efficacy of additional use of prostacyclin analogs for PAH and try to minimize heterogeneity. Further design of RCTs on evaluation of the efficacy of additional use of the intravenous preparations of prostacyclin analogs or the efficacy of prostacyclin analogs in special populations (newborn, children, the elderly, etc.) is still necessary.

## Conclusion

In conclusion, additional prostacyclin analog treatment exerted additional benefits on clinical worsening, exercise capacity, functional class, mPAP, and cardiac index in PAH patients. Furthermore, prostacyclin analogs were relatively safe when added to background targeted therapies, although it was associated with overall risk of adverse events.

## Data Availability

The original contributions presented in the study are included in the article/Supplementary Material, further inquiries can be directed to the corresponding authors.
