# Supplementary material for: Additional Use of Prostacyclin Analogs in Patients With Pulmonary Arterial Hypertension: A Meta-Analysis
Source: Front Pharmacol. 2022 Feb 9;13:817119. doi: 10.3389/fphar.2022.817119 (PMC8864222; doi:10.3389/fphar.2022.817119)
Supplement: Supplementary file 1 [file Table1.DOCX]

Table S1. Search strategy and results.

| **Database** | **search strategy** | **Results (n)** |
| --- | --- | --- |
| PubMed | ("pulmonary arterial hypertension"[Title/Abstract]) AND (prostacyclin[Title/Abstract] OR treprostinil[Title/Abstract] OR iloprost[Title/Abstract] OR beraprost[Title/Abstract] OR epoprostenol[Title/Abstract]) Filters: Clinical Study, Clinical Trial, Humans | 155 |
| EMBASE | 'pulmonary arterial hypertension':ab,ti AND (prostacyclin:ab,ti OR treprostinil:ab,ti OR iloprost:ab,ti OR beraprost:ab,ti OR epoprostenol:ab,ti) AND [embase]/lim AND [humans]/lim AND [clinical study]/lim | 1322 |
| ClinicalTrials.gov | Pulmonary Arterial Hypertension \| prostacyclin OR treprostinil OR iloprost OR beraprost OR epoprostenol | 178 |
