# Supplementary figures and images for: Additional Use of Prostacyclin Analogs in Patients With Pulmonary Arterial Hypertension: A Meta-Analysis
Source: Front Pharmacol. 2022 Feb 9;13:817119. doi: 10.3389/fphar.2022.817119 (PMC8864222; doi:10.3389/fphar.2022.817119)

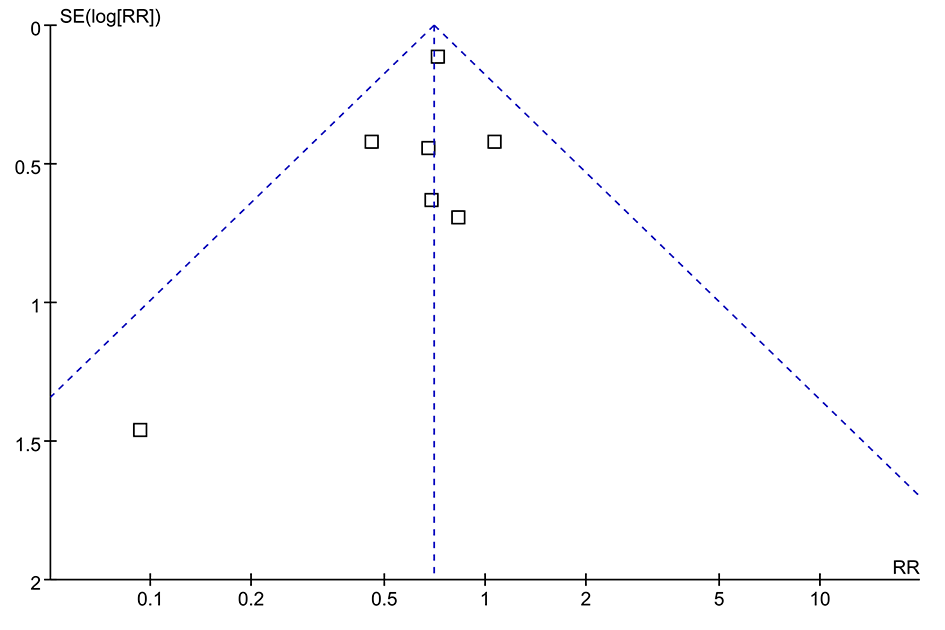

Supplement: Supplementary file 2 [file Image2.TIF]

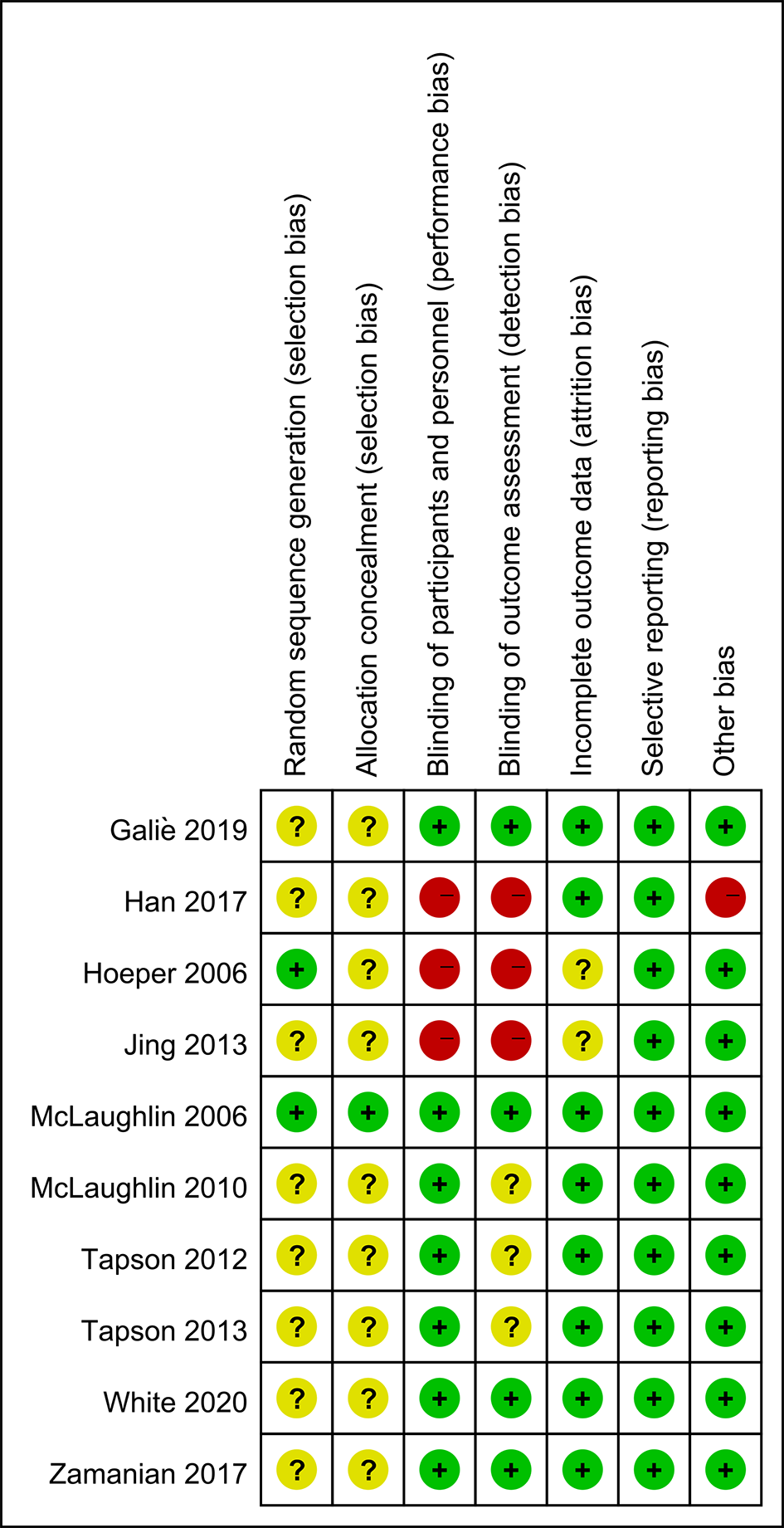

Supplement: Supplementary file 3 [file Image1.TIF]
