# Supplementary material for: Additional Use of Prostacyclin Analogs in Patients With Pulmonary Arterial Hypertension: A Meta-Analysis
Source: Front Pharmacol. 2022 Feb 9;13:817119. doi: 10.3389/fphar.2022.817119 (PMC8864222; doi:10.3389/fphar.2022.817119)
Supplement: Supplementary file 4 [file Table2.DOCX]

Table S2. Results of sensitivity analyses from a meta-analysis of randomized controlled trials.

| Outcome measure | RCTs, n | Summary effect (95% CI) | *p* | *I*^2^, % |
| --- | --- | --- | --- | --- |
| **Double-blind studies only** | | | | |
| Clinical worsening | 5 | RR, 0.72 (0.58, 0.88) | 0.002 | 0 |
| All-cause mortality | 4 | RR, 0.95 (0.55, 1.65) | 0.86 | 0 |
| 6-minute walk distance | 4 | WMD, 13.60 (6.39, 20.81) | < 0.001 | 0 |
| NYHA/WHO functional class | 5 | RR, 1.48 (1.13, 1.94) | 0.004 | 20 |
| Mean pulmonary artery pressure | 1 | WMD, -8 (-11.38, -4.62) | < 0.001 | NA |
| Pulmonary vascular resistance | 1 | WMD, -245 (-372.94, -117.06) | < 0.001 | NA |
| **Studies with a sample size more than 30 only** | | | | |
| 6-minute walk distance | 6 | WMD, 13.92 (6.89, 20.95) | < 0.001 | 17 |
| NYHA/WHO functional class | 5 | RR, 1.57 (1.18, 2.09) | 0.002 | 49 |
| Mean pulmonary artery pressure | 2 | WMD, -5.36 (-10.40, -0.31) | 0.04 | 80 |
| Pulmonary vascular resistance | 2 | WMD, -199.37 (-303.23, -95.52) | < 0.001 | 30 |
| Cardiac index | 1 | WMD, 0.28 (0.04, 0.52) | 0.02 | NA |
| **Published studies only** | | | | |
| 6-minute walk distance | 6 | WMD, 37.70 (1.31, 74.08) | 0.04 | 95 |
| NYHA/WHO functional class | 4 | RR, 1.80 (1.03, 3.15) | 0.04 | 58 |

RCTs, randomized controlled trials; CI, confidence interval.
